# Supplementary material for: Effects of Different Voided Urine Sample Storage Time, Temperature, and Preservatives on Analysis with Multiplex Bead-Based Oncuria Bladder Cancer Immunoassay
Source: Diagnostics (Basel). 2025 Jan 9;15(2):138. doi: 10.3390/diagnostics15020138 (PMC11763964; doi:10.3390/diagnostics15020138)
Supplement: Supplementary file 1 [file diagnostics-15-00138-s001.zip › diagnostics-3352937-supplementary.pdf]

Supplemental Table S1. Mean urinary (±SD) concentrations of 10 biomarkers assessed by Oncuria in control samples

	**Control**	
	Mean	*SD*
MMP9 (pg/mL)	334.0124	13.71452
CXCL8/IL8 (pg/mL)	467.3011	18.34252
VEGFA (pg/mL)	190.8052	9.330939
IX/CA9 (pg/mL)	1.32579	0.144428
SDC1 (pg/mL)	7555.161	1044.38
PAI1 (pg/mL)	497.9924	73.00582
ApoE (pg/mL)	383.6165	92.38655
A1AT (pg/mL)	67634.09	6250.648
ANG (pg/mL)	401.4456	42.05037
MMP10 (pg/mL)	2.241133	0.616335
